# A bacterial quercetin oxidoreductase QuoA-mediated perturbation in the phenylpropanoid metabolic network increases lignification with a concomitant decrease in phenolamides in *Arabidopsis*

**DOI:** 10.1093/jxb/ert310

**Published:** 2013-10-01

**Authors:** Sheela Reuben, Amit Rai, Bhinu V. S. Pillai, Amrith Rodrigues, Sanjay Swarup

**Affiliations:** Department of Biological Sciences, National University of Singapore, 14, Science Drive 4, Singapore 117543

**Keywords:** Anthocyanins, auxotrophic mutant, branch points, flavonoids, lignin pathway, metabolomics, plant-growth-promoting rhizobacteria, PGPR, *tt6*.

## Abstract

Metabolic perturbations by a gain-of-function approach provide a means to alter steady states of metabolites and query network properties, while keeping enzyme complexes intact. A combination of genetic and targeted metabolomics approach was used to understand the network properties of phenylpropanoid secondary metabolism pathways. A novel quercetin oxidoreductase, QuoA, from *Pseudomonas putida*, which converts quercetin to naringenin, thus effectively reversing the biosynthesis of quercetin through a *de novo* pathway, was expressed in *Arabidopsis thaliana*. *QuoA* transgenic lines selected for low, medium, and high expression levels of *QuoA* RNA had corresponding levels of QuoA activity and hypocotyl coloration resulting from increased anthocyanin accumulation. Stems of all three *QuoA* lines had increased tensile strength resulting from increased lignification. Sixteen metabolic intermediates from anthocyanin, lignin, and shikimate pathways had increased accumulation, of which 11 paralleled *QuoA* expression levels in the transgenic lines. The concomitant upregulation of the above pathways was explained by a significant downregulation of the phenolamide pathway and its precursor, spermidine. In a *tt6* mutant line, lignifications as well as levels of the lignin pathway metabolites were much lower than those of *QuoA* transgenic lines. Unlike *QuoA* lines, phenolamides and spermidine were not affected in the *tt6* line. Taken together, these results suggest that phenolamide pathway plays a major role in directing metabolic intermediates into the lignin pathway. Metabolic perturbations were accompanied by downregulation of five genes associated with branch-point enzymes and upregulation of their corresponding products. These results suggest that gene–metabolite pairs are likely to be co-ordinately regulated at critical branch points. Thus, these perturbations by a gain-of-function approach have uncovered novel properties of the phenylpropanoid metabolic network.

## Introduction

Perturbations can be caused in metabolic networks to uncover relationships that exist between different parts of the networks. While physiological or environmental perturbations can be used to study such relationships, they can affect different points in multiple pathways. Genetic approaches, on the other hand, have been used to cause perturbations of specific steps in targeted metabolic pathways. These perturbations can be induced via loss-of-function approaches such as gene deletions (Franke *et al.*, 2002) or silencing (Besseau *et al.*, 2007). In comparison with these approaches, expression of plant or bacterial enzymes in transgenic plants provides a means for perturbing metabolic pathways by gain-of-function methods. This approach has been effective in introducing new functions to increase the accumulation of desired compounds or to reroute metabolites. Expression of bacterial enoyl-CoA hydratase/lyase, for example, results in rerouting in the phenylpropanoid pathway and leads to depletion of phenolics and accumulation of vanillic acid (Mayer *et al.*, 2001). During perturbation of the isoprenoid pathway by the expression of a bacterial gene encoding 1-deoxy-d-xylulose-5-phosphate synthase in transgenic potato tubers, metabolic channelling of *trans*-zeatin riboside and carotenoids increases 2-fold and levels of phytoene increase 6- to 7-fold (Morris *et al.*, 2006).

Phenylpropanoids are secondary metabolites derived from phenylalanine. They play a critical role in signalling and plant defence against abiotic or biotic stresses. Phenylpropanoid-based polymers in plants, such as lignin, suberin, or condensed tannins, contribute to the physical stability and robustness towards environmental damages from drought or wounding. Metabolic intermediates from the upper phenylpropanoid biosynthesis pathway serve as starting point for multiple pathways connected by branches to form flavonoids, anthocyanins, proanthocyanins, lignins, and phenolamides. Together, all these pathways constitute the phenylpropanoid metabolic network. A plethora of phenylpropanoid biosynthetic mutants have facilitated the identification and functional characterization of genes and enzymes of the phenylpropanoid metabolic network. Among these, several enzymes of the flavonoid pathway, such as chalcone synthase (CHS), chalcone-flavanone isomerase (CHI), and dihydroflavonol 4-reductase (DFR), form a complex in order to regulate the partitioning of intermediates among competing pathways and determine the intracellular deposition of end-products (Burbulis and Winkel-Shirley, 1999; Rasmussen and Dixon, 1999; Winkel, 2004). These enzyme complexes play an important role in channelling of intermediates, limiting their diffusion into the surrounding milieu, which determines metabolic network properties under different conditions. Hence, keeping such complexes intact, while perturbing metabolic pathways in order to estimate network behaviour, would be an ideal approach. Given the highly complex nature of phenylpropanoid metabolic network and the importance of its constituent metabolites, it makes an ideal target to decipher the relationships among the pathways of this network.

**Fig. 1.**
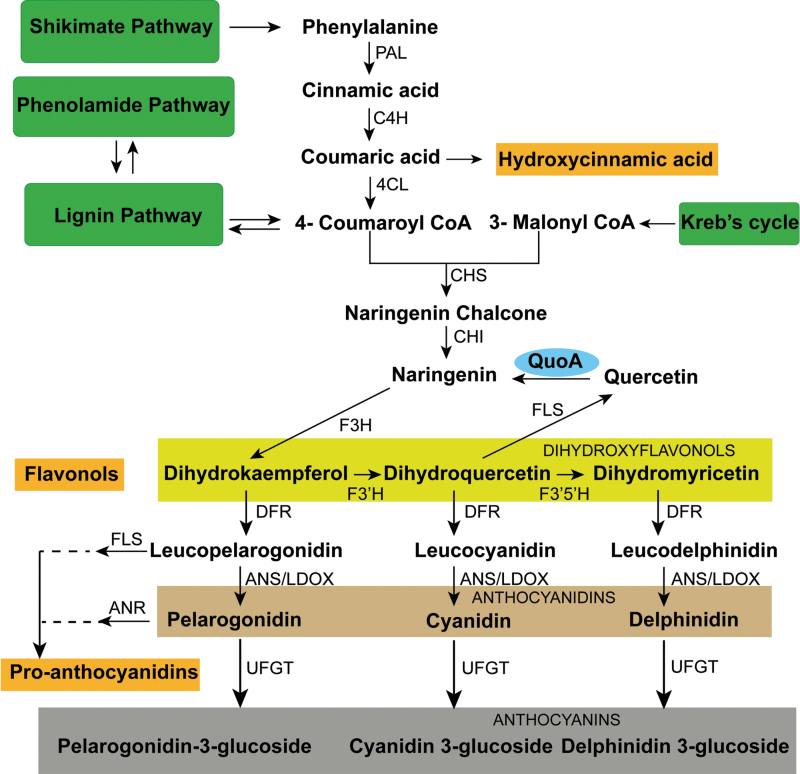
Phenylpropanoid metabolic network map in *Arabidopsis.* PAL, phenylalanine ammonia lyase; C4H, cinnamate 4-hydroxylase; 4CL, 4-coumaroyl-coenzyme A ligase; CHS, chalcone synthase; CHI, chalcone-flavanone isomerase; F3H, flavanone 3-hydroxylase; F3′H, flavonoid 3′-mono-oxygenase; DFR, dihydroflavonol 4-reductase; QuoA, quercetin oxidoreductase; FLS, flavonol synthase; F3′5′H, flavonoid 3′5′-hydroxylase; LAR, leucoanthocyanidin reductase; LDOX, leucoanthocyanidin dioxygenase; BAN, banyuls; ANR, anthocyanidin reductase; ANS, anthocyanidin synthase; LDOX, leucoanthocyanidin dioxygenase; FGT, flavonoid glycosyl transferase; C3H, coumarate-3-hydroxylase; F5H, ferulate-5-hydroxylase; CCR1, cinnamoyl-CoA reductase 1; OMT1, flavonol 3′-*o*-methyltransferase; SGT, sinapate-1-glucosyltransferase; SNG1, sinapoylglucose:malate sinapoyltransferase; HCT, hydroxycinnamoyl-CoA shikimate/quinate hydroxycinnamoyl transferase; CAD, cinnamyl alcohol dehydrogenase; CCoAOMT, caffeoyl CoA *o*-methyltransferase; UFGT, UDP-flavonol glucose transferase; SHT, spermidine hydroxycinnamoyl transferase; bHLH, basic helix–loop–helix; WDR, WD-repeat protein with WRKY domains; TT, transparent testa; TTG1, transparent testa glabra 1. (This figure is available in colour at *JXB* online.)

We previously reported a *Pseudomonas putida* strain PML2 capable of utilizing quercetin as a sole carbon source for growth and elucidated a catabolic pathway for quercetin degradation in this strain using a comparative metabolomics approach (Pillai and Swarup, 2002). The first step in this pathway is the conversion of quercetin to naringenin involving dehydroxylation at two sites. The corresponding gene has been designated *QuoA*. As phenylpropanoid biosynthesis in plants involves the formation of quercetin from naringenin, we envisaged that *QuoA* expression in plants would provide us with a genetic tool to ‘reverse’ this biosynthetic step using a gain-of-function approach and would enable us to monitor the perturbational effects in the metabolic networks (Fig. 1).

Here, we report the enzyme activity of QuoA oxidoreductase in transgenic *Arabidopsis* and describe metabolic changes in the phenylpropanoid pathways and their resulting phenotypes in the transgenic lines that were selected based on varying levels of *QuoA* expression. Significant rerouting of metabolites was observed in multiple branched pathways in association with specific changes in gene expression of biosynthetic enzymes and their associated metabolites. The phenylpropanoid metabolic network therefore seems to be regulated at multiple levels in a co-ordinated manner.

## Materials and methods

### Bacterial strains, plasmids, plant stocks, and growth conditions

Bacterial strains and plasmids used in this study are listed in Supplementary Table S1 at *JXB* online. *Escherichia coli* cultures were grown at 37 °C in Luria–Bertani medium. All *P. putida* cultures were grown in Stanier’s mineral salts as described previously (Pillai and Swarup, 2002) by supplementing with appropriate carbon sources. *Agrobacterium* cultures were grown at 28 °C. When required, the antibiotics (Sigma Chemical Co., St Louis, MO, USA) chloramphenicol, gentamycin, kanamycin, ampicillin, rifampicin, and tetracycline were added to final concentrations of 30, 10, 15, 100, 10, and 30 µg ml^–1^, respectively.

Seeds of the isogenic *tt6* mutant and Landsberg WT plants were obtained from the *Arabidopsis* seed stock centre (Arabidopsis Biological Resource Center, USA). Plants were grown following standard protocols (Narasimhan *et al.*, 2003) with a 16h photoperiod and at 21–23 °C either in growth chambers or in growth rooms. For anthocyanin analysis, seedlings were grown for 6 d in Murashige and Skoog medium with sucrose, and for metabolite extraction and quantitative PCR studies they were grown for 35 days in sterilized potting mix.

### Chemicals and reagents

High-performance liquid chromatography (HPLC)-grade acetonitrile and methanol (Fisher Scientific, UK) were used for all experiments. The analytical reagents hydrochloric acid, glacial acetic acid, and sodium hydroxide were from Amersham Pharmacia Biotech (Uppsala, Sweden). Quercetin and naringenin were obtained from Sigma Chemical Co. Flavonoid stock solutions were prepared as described previously (Pillai and Swarup, 2002).

### Creation of *QuoA* transgenics

The coding sequence for *QuoA* was amplified from the wild-type (WT) microbial strain PML2 using forward primer with an *Nco*I restriction endonuclease site (QuoA-5′*Nco*I: CATGUC CATGGUAAGCAGCCAAACC-3′) and a reverse primer with a *BamH*I site (QuoA-3′*Bam*: 5′CGUGGATCCUTCAGCGCGGA ATGAC-3′) to create the expression cassette pGB*QuoA* in pGB1 (Bloemberg *et al.*, 1997). PCR amplification parameters were as follows: initial denaturation at 95 °C for 1min; 69 °C for 30 s; repeat for 6 cycles; 95 °C for 30 s; 67 °C for 3min; repeat cycling 31 times; and a final extension at 68 °C for 10min. The 2.2kb PCR-amplified *QuoA* coding sequence DNA from pGB*QuoA* was cloned into pRTL-2GUS to replace the β-glucuronidase (GUS) gene, yielding pRTL*QuoA.* An ~3.6kb *Hind*III fragment from pRTL*QuoA* was cloned into the binary vector pBIN20 (Hennegan and Danna, 1998; Supplementary Table S1) resulting in the ~15.8kb clone pBin*QuoA* with *QuoA* under the control of a cauliflower mosaic virus 35S promoter. *Agrobacterium* AGL1 containing pBin*QuoA* was used to transform *Arabidopsis* Columbia (Col-0) plants using the floral-dip method (Clough and Bent, 1998) and other standard protocols used for *Arabidopsis* available from The *Arabidopsis* Information Resource (TAIR, http://www.arabidopsis.org/, 10 September 2013). Plants were grown under long days (8h dark) until they flowered. Dry seeds were harvested after floral dipping. From nearly 1000 seeds, kanamycin-resistant seeds were identified and 16 independent T3 generation lines were established. The transformed lines were confirmed by genomic DNA PCR using the *QuoA* gene-specific primers QuoA1 5′-CAGCCGGCGTCGAGTTCTT-3′ and QuoA2 5′-GACTCGATGATCAACCGCT-3′ under the conditions described above.

### QuoA enzyme activity in transgenic *Arabidopsis*

Plant crude protein extracts were obtained by grinding 5-week-old plants in liquid nitrogen followed by the addition of 0.1M phosphate buffer (pH 7.0) containing 1mM dithiothreitol and 0.2mM phenylmethylsulfonyl fluoride. The extract was partially purified using a Sepharose-25 column to remove endogenous co-factors and small molecules <5000Da. The enzyme assay was performed with 5mM substrate quercetin, 25 µl of crude enzyme preparation, 200 µM β-NAD^+^ and 400 µM ferrous ammonium sulfate. The reaction mixture was incubated at 30 °C and the reaction stopped after 1h by adding proteinase K (2mg ml^–1^). The products were analysed by electrospray ionization mass spectrometry (ESI-MS) for the presence of naringenin.

### RNA level analyses

Total RNA was extracted from the 5-week-old transgenic plants (*Q*2-17) or WT Columbia lines using Trizol™ reagent (Invitrogen) and semi-quantitative reverse transcription PCR analysis carried out as per the manufacturer’s instructions. The PCR parameters used were 95 °C for 5min, followed by 30 cycles of 95 °C for 1min, and an annealing temperature for the *QuoA* primers QuoA1 and QuoA2 of 56 ° C and for the tubulin primers of 58 ° C for 2 and 1min for the *QuoA* and tubulin primers, respectively, at 72 °C. The final extension time was 10min at 72 °C. For the different biosynthetic genes and the regulatory genes, gene-specific primers were used as listed in Supplementary Table S2 at *JXB* online. Real-time quantitative PCR was performed on an ABI Prism 7700 thermal cycler using SYBR Green (Qiagen, USA). RNA concentrations were determined using a Nanodrop^TM^ (Nanodrop Technologies, USA). β-Tubulin primers were used as a control in each experiment. The comparative *C*_T_ method (ΔΔ*C*_T_) for relative quantification of gene expression was used for calculating the fold change. The primers used for biosynthetic and regulatory genes are given in Supplementary Table S2. All amplifications were done with three biological replicates.

### Anthocyanins and metabolite extraction

For extraction of anthocyanins, 6-day-old seedlings were used, as anthocyanin accumulation is reportedly very high during this stage (Pelletier *et al.*, 1999; Peer *et al.*, 2001). Seedlings (0.1g) were ground in 0.2ml of acidified methanol (1%, v/v, concentrated HCl) and stored at 4 °C overnight to facilitate the total extraction of anthocyanins. Three such biological replicates were analysed. The suspension was centrifuged at 25 000*g* for 20min. Absorbance was read at 530nm using a Beckman Spectrophotometer DU 640 B (Beckman Coulter, USA). Relative anthocyanin units were calculated as *A*=*A*_530_ – 1/4×*A*_650_ g^–1^ of fresh weight.

For metabolite extraction, three randomly selected plants (35 days old) from each group were taken as one replicate sample. Three such replicates were analysed for each experiment. Adult plants were used here to study the lignin content as these accumulate in older plants. Plants were ground with liquid nitrogen and extracted with 80% methanol. Methanol (100 µl of 80%) was added to every 0.1g of tissue, and extracts were centrifuged at 25 000*g* at 4 °C for 30min. The supernatant was further filtered through a syringe-tip polytetrafluoroethylene filter (0.2 µm) prior to use.

### Stem stiffness test

Thirty-five-day-old *Arabidopsis* plants were used to test tensile strength. Stems were measured for cross-sectional area, length, and thickness. Stems were then placed from both ends to two fixed ends of a tensile strength tester (5900 Series Mechanical Testing Systems; Instron, USA). The displacement speed for the two ends was fixed to 0.5mm min^–1^. Maximum load and extension at breaking point were measured and the modulus calculated as stress over strain in MPa using instruments and software provided by the manufacturer.

### Assay of Klason lignin content

Lignin content was determined by Klason method as described by Coleman *et al.* (2008). The second internodes of stems (from rosette leaves) of 5-week-old transgenic *Arabidopsis* lines were harvested and the internodes were then cut by hand into thin sections before transferring them into an 80 °C oven. The dried stem sections were ground to a ﬁne powder with a mortar and pestle, washed four times in methanol, and then dried again to remove the methanol. Dried extract (200mg) was mixed with 5ml of 72% (w/w) sulfuric acid at 30 °C and hydrolysed for 1h. The hydrolysate was diluted to 3% sulfuric acid and autoclaved for 1h at 121 °C. The solid residue was ﬁltered through a glass ﬁlter. Finally, samples were dried at 80 °C overnight and then weighed again. The lignin content was measured and expressed as a percentage of the original weight of cell-wall residue (Dence, 1992).

### Liquid chromatography and mass spectrometry analysis

Metabolite extracts from transgenic plants, *tt6*, Landsberg (Ler), and Col-0 *Arabidopsis* plants were analysed by offline HPLC-MS.The chromatographic analysis was performed on a Jupiter C18 360Å 5 µm reverse phase column (Jupiter Proteo^TM^, 4.6×250mm; Phenomenex, CA, USA). The HPLC system used was an Akta Purifier 10 (Amersham Biosciences, UK). HPLC and MS parameters were as described previously (Pillai and Swarup, 2002), with minor modifications as described here. The buffers used were pH 3 water acidified with glacial acetic acid (buffer A) and 100% acetonitrile (buffer B). The metabolites were eluted with buffer B. The gradient was set as follows: 0–10% (v/v) B (4min), 10–30% (v/v) B (16min), 30–50% (v/v) B (20min), 50–100%(v/v) B (28min), 100% (v/v) B (40min), and 100–10%(v/v) B (46min). The metabolites were detected using three UV wavelengths, 236, 255, and 215nm. Prior to the next injection, the column was equilibrated for 30min with 10% buffer B. A 100 µl aliquot of each sample was manually injected and the flow rate was maintained at 0.4ml min^–1^; individual fractions showing peaks from HPLC were collected and analysed by TQ Xevo^TM^ MS (Waters Corporation, MA, USA), a tandem quadruple mass spectrometer. The electrospray probe was operated in positive mode with capillary voltage at 0.5kV and cone voltage at 40V. Source temperature and desolvation temperature were set at 150 and 600 °C with desolving gas flow rate at 1200 l h^–1^ and collision gas (argon) flow rate at 0.18ml min (4×10^–3^ mbar). Ramp linear collision energy was applied for MS/MS analysis with collision energy ranging between 15 and 45eV. The injection volume was 10 µl, and scans in function were 42. Sample analysis was performed in triplicate (three biological samples and three technical samples). MassLynx software version 4.1 (Waters Corporation) was used to control the instrument and calculate accurate masses. Data was normalized with respect to lysine as its level was not affected by QuoA in all the lines used in this study (Supplementary Tables S3 and S4 at *JXB* online) and the log_2_ values of normalized intensities were calculated with respect to WT. The coefficient of variation of lysine levels among the lines was <0.5% compared with those of the differential metabolites, which were higher by at least two orders of magnitude. For the transgenics, Col-0 was used for comparison, and the loss-of-function mutant *tt6* was compared with its mutant background Ler. The metabolites were identified either by commercial standards or by comparing fragment ions with the MS/MS profile of compounds submitted in MassBank (Horai *et al.*, 2010), listed in Supplementary Table S5 at *JXB* online. The standards used in this study and their MS/MS peaks are listed in Supplementary Table S6 at *JXB* online.

Quantification of metabolites was performed in multiple reaction monitoring (MRM) mode by collision-induced dissociation tandem mass spectrometry (QTrap4000; ABSciex, MA, USA) in the positive mode (capillary voltage 4500V, declustering potential 100V). At least two fragment ions for each metabolite were used for quantification (Supplementary Table S5). The instrument was set up to cycle through one full-scan mass spectrum (enhanced MS, with Q0 trapping activated) followed by MRM transitions with total cycle time of ~11.6 s (1.1 s per enhanced MS scan, followed by 300 MRM scans of 35ms each). Q1 resolution was set to high (resolution=2500, full width at half maximum=0.4Da at *m*/*z*=1000) and Q3 resolution was set to unit (resolution=1700, full width at half maximum=0.6Da at *m*/*z*=1000).

## Results

### *Pseudomonas* QuoA oxidoreductase converts quercetin to naringenin in transgenic *Arabidopsis* plants

**Fig. 2.**
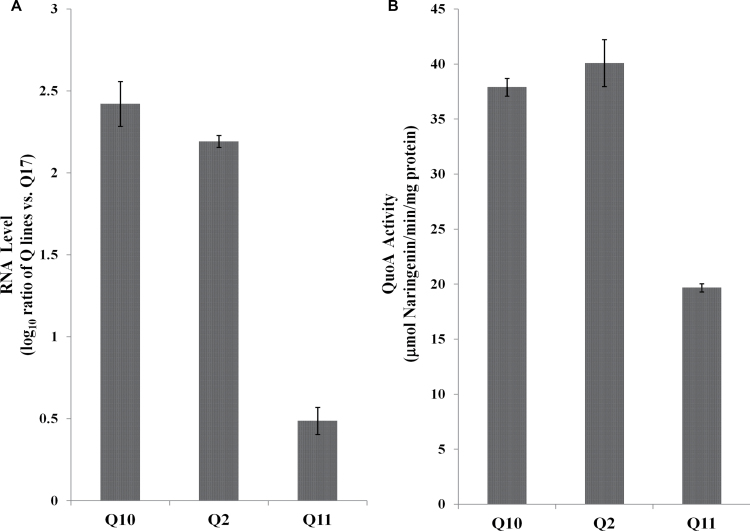
Expression and activity of *P. putida* PML2 *QuoA* transgene in *Arabidopsis* 5-week-old plants. (A) Levels of *QuoA* transcripts measured by quantitative real-time PCR in selected independent transgenic plants. Fold change values were normalized based on *QuoA* RNA levels in Q17, which expresses *QuoA* at low levels. All *QuoA* RNA levels were significantly different from each other (*P*<0.001). (B) *QuoA* activity in crude protein extract from transgenic lines measured by quantifying levels of naringenin produced by the substrate quercetin. Partially purified protein extract from transgenic plants was incubated with quercetin and the reaction mix. Naringenin levels were measured by ESI-MS and conversion rates were calculated per unit time incubation mg^–1^ of protein. Basal conversion rates were corrected based on Col-0. All *QuoA* activity levels were significantly different from each other (*P*<0.01). Data presented are means±SEM from two independent experiments each with five biological replicates.

In order to perturb the phenylpropanoid biosynthetic pathway in a model plant, 16 gain-of-function lines of *Arabidopsis thaliana* were created by introducing the gene *QuoA*, encoding an oxidoreductase from the plant-growth-promoting rhizobacterial strain *P. putida* PML2 that converts quercetin to naringenin (Pillai and Swarup, 2002). All 16 independently transformed F4 lines (named *Q*2–*Q*17) were confirmed to express *QuoA* by semi-quantitative reverse transcription PCR, and a subset of these lines was used to quantify RNA levels by quantitative real-time PCR. As expected, WT plants showed no detectable transcripts of *QuoA*. RNA levels of *QuoA* in transgenic lines were normalized against that of *Q*17, which had the lowest expression. We selected *Q*10, *Q*2, and *Q*11 as high, moderate, and low *QuoA* expression lines, respectively (Fig. 2A). *Q2* had marginally lower mean expression levels than *Q10*, which was statistically significant. These three lines with varying transgene expression levels were used for further studies to associate metabolic and other phenotypes with perturbational effects.

To test QuoA activity in transgenic plants, crude protein extracts from the three transgenic lines and Col-0 were partially purified and assayed for oxidoreductase activity. Accumulation of naringenin as a product of QuoA activity was quantified per unit time mg^–1^ of protein, using the MRM mode of MS. Basal conversion rates were corrected based on extracts from WT Col-0 (11.5 µmol naringenin min^–1^ mg^–1^ of protein). The conversion rate of quercetin to naringenin by QuoA was high in *Q*10 and *Q*2, while it was nearly half of *Q*10 level in *Q*11 (Fig. 2B). QuoA activity was, therefore, consistent with its expression levels in the transgenic lines in converting quercetin to naringenin, thus ‘reversing’ the biosynthesis of quercetin from naringenin that occurs in WT plants.

### QuoA expression results in increased coloration of hypocotyls and increased stem stiffness

**Fig. 3.**
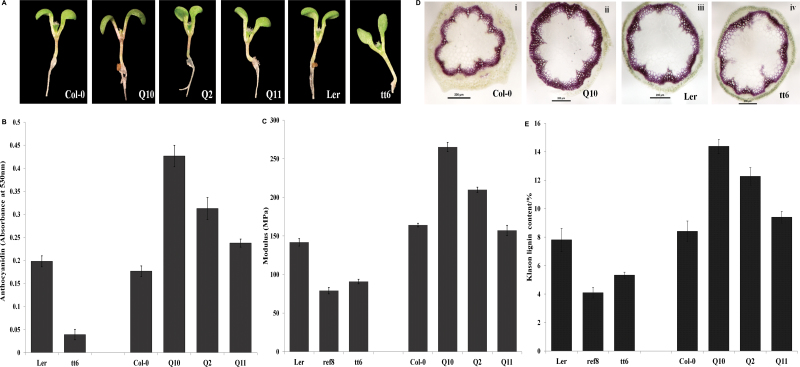
Phenotypes associated with *QuoA* transgene expression in transgenic lines. (A) *QuoA* transgenic seedlings showing accumulation of anthocyanidins. The *tt6* mutant, which does not produce anthocyanidins, was used as a control. (B) Anthocyanidin quantification in 6-day-old *QuoA* transgenic seedlings. Anthocyanidins were extracted using acidified methanol and absorbance was measured at 530nm. WT Col-0 and *tt6* seedling are shown as controls. All experiments were repeated three times, and results are shown as mean±SEM based on three replications (all significant at *P*<0.01). (C) Young’s modulus of stem internodal segments. Plants (*n*=5) were subjected to a displacement speed of 0.5mm min^–1^ until the stem reached their break point in a tensile tester. Maximum load and extension at breaking point were measured and modulus calculated as stress over strain in MPa. The results are shown as means±SEM based on nine replications (all significant at *P*<0.0001). (D) Phluoroglucinol staining of lignin in stem cross- sections of WT Columbia (Col-0), *QuoA* transgenics (Q10), WT Landsberg (Ler), and the *tt6* mutant. Infloresence stems from 5-week-old QuoA transgenic lines were sectioned and stained with phloroglucinol/HCl. All images were taken at ×80 magnification under a dissection microscope with bright-field illumination. (E) Klason lignin content analysis of 5-week-old *Arabidopsis* transgenic stems. The results are shown as means±SEM based on five replications (all significant at *P*<0.01).

In the *Arabidopsis* flavonoid pathway, naringenin is converted to quercetin via the intermediate dihydrokaempferol by flavanone-3-hydroxylase (F3H) encoded by *tt6*. As this affected step is at the junction of upper and lower phenylpropanoid pathways (Fig. 1), we expected that the perturbation would have effects in both directions. A lower phenylpropanoid pathway leads to anthocyanin production, which gives a characteristic colour to various plant parts. Indeed, *QuoA* transgenic seedlings had distinct purple coloration in their hypocotyls (Fig. 3A), which indicated anthocyanin accumulation. Transgenic lines *Q*10 and *Q*2 showed high and moderate levels of anthocyanin accumulation in the hypocotyl region, respectively, while *Q*11 had a similar level of anthocyanins as Col-0. Levels of extracted anthocyanins in *Q*10 were more than double, while *Q*2 had 70% higher anthocyanin than Col-0 (Fig. 3B). Hence, anthocyanin levels were of the same order of magnitude in the transgenic lines as their *QuoA* RNA and enzyme activity levels, respectively. The F3H mutant line *tt6* had no observable coloration (Fig. 3A). As F3H is involved in the formation of dihydrokaempferol from naringenin, which leads to the formation of quercetin in biosynthetic pathways, we used the F3H mutant line *tt6* to compare perturbation with *QuoA* transgenic lines.

Having described colour of hypocotyls as an indicator of perturbation in the lower phenylpropanoid pathways, we investigated phenotypic indicators of perturbation in the lignin pathway, which originates from the upper phenylpropanoid pathway. We focused on the effects on the lignin pathway for two reasons. First, in the upper phenylpropanoid pathway, the lignin branch originates very closely from the point of perturbation by QuoA, and secondly, because previous reports (Besseau *et al.*, 2007; Vanholme *et al.*, 2008; Li *et al.*, 2010) have established close relationship between the flavonoid and lignin pathways. While *Arabidopsis* is not a woody plant, its lignification has been shown to affect stiffness of stem internodes (Zhong *et al.*, 2000; Allen *et al.*, 2009). We therefore tested the mechanical strength of three transgenic lines by performing a stress test on 35-day-old internodal stem segments of Col-0, *Q*10, *Q*2, *Q*11, ref8, *tt6*, and WT Ler plants. Young’s modulus of stem internodal segments, which represents stiffness of the stem, was calculated. The tensile strength of lines decreased in the following order, *Q*10>*Q*2>Col-0>*Q*11>Ler>*tt6*>ref8 (Fig. 3C). As expected, hydroxycinnamoyl-CoA (HCT) mutant ref8, which has highly reduced lignin metabolites, had the lowest tensile strength (Franke *et al.*, 2002). The tensile strength of the three transgenic lines followed the same order as *QuoA* expression and enzymatic activity. In order to confirm that the increase in stiffness was due to lignin accumulation, we stained internodal sections using phloroglucinol/HCl for lignin in the high expression line *Q*10. The lignin staining area was visibly larger in *Q*10 compared with WT Col-0 (Fig. 3D). Lignin levels also showed the same order of Q10>Q2>Col-0>Q11>Ler>tt6>ref8 as tensile strength (Fig. 3E).

As QuoA acts at the flavonoid pathway step involving F3H, we used F3H mutant line, *tt6* to compare its effect with *QuoA* transgenic lines. Metabolic perturbations due to expression of *QuoA* in gain-of-function transgenic lines were expected to be present in both upper and lower phenylpropanoid pathways. In comparison with QuoA lines, which allow changes to occur in both upper and lower phenylpropanoid pathways, metabolic mutant *tt6* was expected to block the lower pathway, resulting in perturbation of only the upper pathway. Stiffness of *tt6* stems was significantly lower while the lignin staining area was smaller than in Ler (Fig. 3C, D). In order to identify which branched pathways could account for increased lignin in *Q* lines compared with *tt6*, we quantified metabolite intermediates from the three linked pathways: the phenylpropanoid, lignin, and phenolamide pathways.

### *QuoA* perturbation leads to increases in lignin and anthocyanin levels and a decrease in levels of phenolamides

**Fig. 4.**
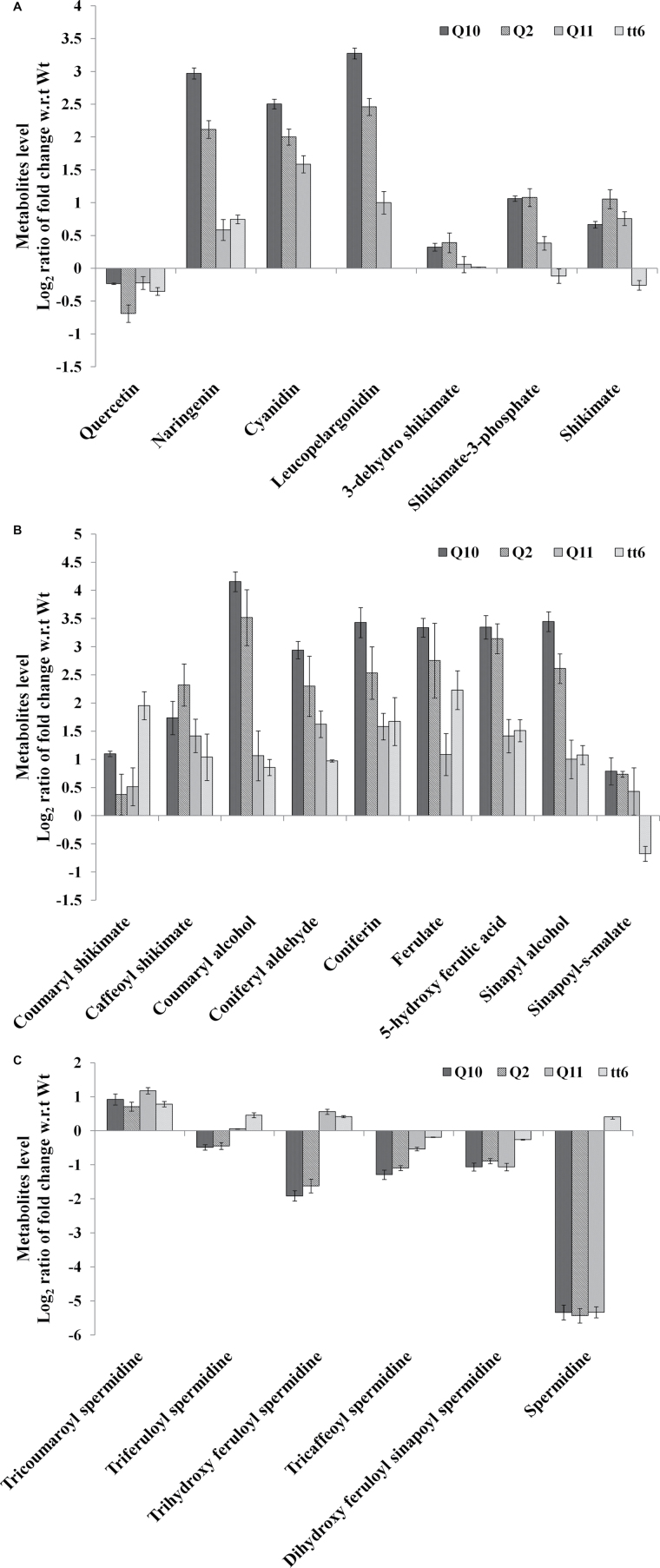
Levels of metabolites from the anthocyanin, lignin, and phenolamide pathways in 5-week-old plants. (A) Relative levels of anthocyanin pathway metabolites in the transgenic lines. (B) Relative levels of lignin pathway metabolites in the transgenic lines. (C) Relative levels of phenolamide pathway metabolites in the transgenic lines. Fold change of metabolites of *QuoA* transgenic lines was calculated with respect to WT Col-0 and of *tt6* with respect to WT Ler, and was plotted as log_2_ values. Multiple-ion monitoring for these metabolites were performed using three fragment ions of each metabolite with maximum intensity. Pure standards were used to select fragment ions for quantification. Data presented are means±SEM from two independent experiments each with five biological replicates (all significant at *P*<0.001).

Metabolite levels from the phenylpropanoid, phenolamide, and lignin pathways were quantified by MRM in the three *QuoA* transgenic lines *Q*2, *Q*10, and *Q*11. Steady-state levels of quercetin were reduced by 1.5- to 2-fold and naringenin levels were increased by 4- to 7-fold in transgenic lines compared with WT. This suggested conversion of quercetin to naringenin by *QuoA* in the transgenic lines. Metabolites from the lower phenylpropanoid pathways increased in all transgenic lines. Hence, the levels of metabolites from both upper and lower pathways from the point of perturbation were significantly affected. Levels of cyanidin and leucopelargonidin increased 4- to 9-fold in the transgenic lines (Fig. 4A), with the highest fold change in *Q*10 compared with other transgenic lines, which is consistent with our observation of anthocyanin accumulation in transgenic lines.

Among the metabolites from the upper phenylpropanoid pathway, 4-coumaryl-CoA showed a 1.6-fold increase in transgenic lines when compared with Col-0. As the upper phenylpropanoid pathway is connected to the lignin metabolic pathway via 4-coumaryl-CoA, we tested perturbational effects in lignin metabolic pathways. End-products and a few intermediates of the lignin metabolic pathways showed increased accumulation. Caffeoyl shikimate, coniferyl aldehyde, ferulate, 5-hydroxy ferulic acid, and end-product coniferin (Fig. 4B) showed 4- to 10-fold increased accumulation in the transgenic lines, with *Q*10 showing higher accumulation compared with other transgenic lines. The highest change was that of coumaroyl alcohol with a 2- to 11-fold increase. As 4-coumaryl-CoA was the only metabolite from the upper phenylpropanoid pathway with increased levels, we hypothesized the involvement of other possible pathways linked to lignin pathways that might be affected by the *QuoA*-introduced perturbation. We therefore tested intermediates of the recently described phenolamide pathway, which is linked to lignin pathways (Matsuno *et al.*, 2009). All the metabolites of the pathways except tricoumaroyl spermidine were decreased to significantly lower levels (Fig. 4C) compared with WT. Spermidine levels showed a decrease by 64-fold in comparison with WT.

### Lignin pathway intermediates increase but phenolamide pathway metabolite levels are not affected in the flavanone-3-hydroxylase mutant *tt6* line

In comparison with *QuoA* expression lines, the mutant *tt6* line showed an absence of anthocyanins, as expected (Fig. 3A, B). Cyanidin and leucopelargonidin were also not detected in the *tt6* mutant line. This effect is probably due to the blockage of lower phenylpropanoid pathways brought about by loss of F3H function.

The *tt6* mutant line had a marginal increase in naringenin level by 1.3-fold, which is probably due to the leaky nature of the *tt6* mutation. Metabolic changes in the lignin pathway were not as high as in gain-of-function lines, although the same trend of increased metabolite levels was observed; for example, ferulate and coniferin levels increased by 3- to 4-fold in *tt6* (Fig. 4B). Several lignin metabolites such as coumaryl alcohol, coumaraldehyde, and sinapyl alcohol showed 1.3- to 2-fold increases in concentration when compared with Ler. In *tt6*, levels of phenolamide pathway metabolites were not significantly affected (Fig. 4C).

### Perturbation by QuoA leads to changes in expression of structural genes associated with branch points of metabolic networks

**Fig. 5.**
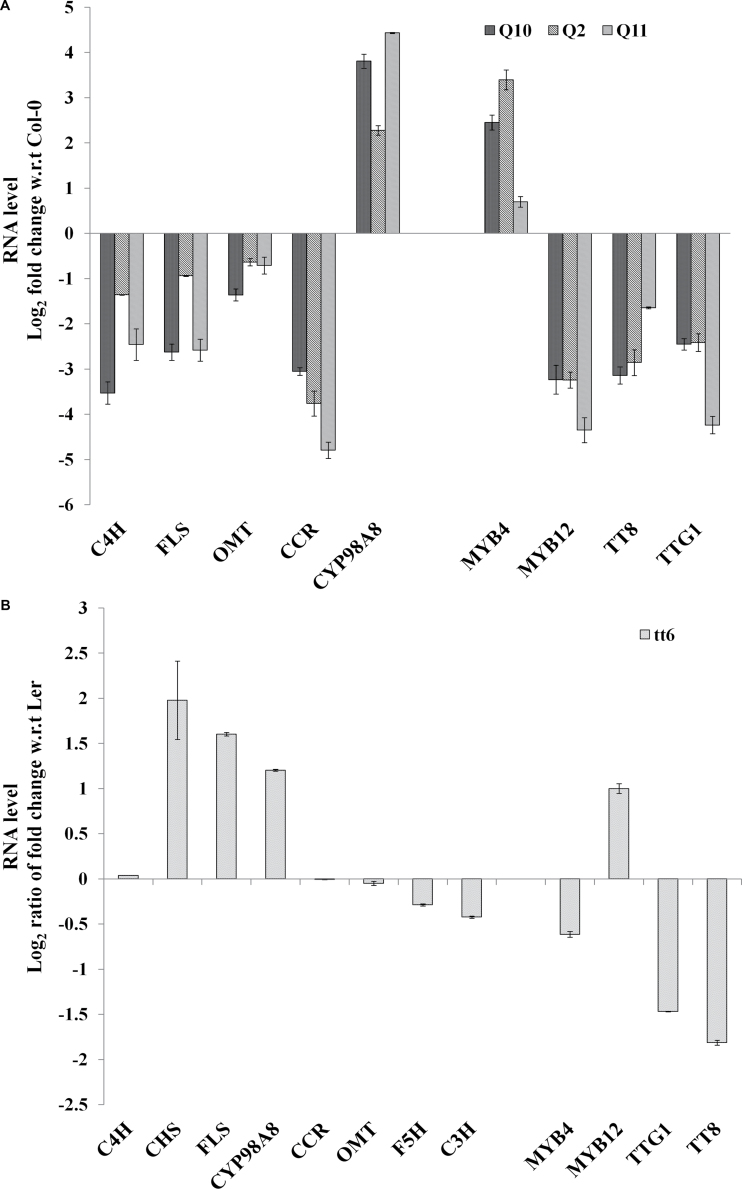
RNA levels of selected structural genes in 5-week-old *QuoA* transgenic lines and the *tt6* mutant. (A) RNA levels of structural genes in *QuoA* transgenic plants. (B) RNA levels of structural genes in the *tt6* mutant. Real-time quantitative PCR was performed and *C*_T_ values were normalized using tubulin as a control. Fold changes were calculated based on the ΔΔ *C*_T_ method. *MYB4*, *MYB12*, *TTG1*, and *TT8* are regulators of phenylpropanoid pathway*. C4H*, cinnamate hydroxylase; *FLS*, flavonol synthase; *CYP98A8*, phenolamide pathway gene; *CCR*, cinnamoyl-CoA reductase; *OMT*, flavonol 3′-*o*-methyltransferase. Results are shown as means±SEM based on three replications (all significant at *P*<0.05) and experiments were repeated twice for all genes.

As metabolite levels for multiple pathways were affected, we questioned whether some of these resulted from gene expression changes. We systematically compared the RNA levels for genes at each step in the flavonoid and anthocyanin biosynthetic pathways (eleven genes) and for key genes in the lignin pathway (six genes). Expression levels of five genes, namely cinnamate-4-hydroxylase (*C4H*) and flavonol synthase (*FLS*) genes in the flavonoid pathway, a cytochrome P450 (*CYP98A8*) from the phenolamide pathway, and cinnamoyl-CoA reductase (*CCR*) and flavonol 3′-*o*-methyltransferase (*OMT*), genes in the lignin pathway, showed differential expression in *QuoA* lines compared with Col-0 levels (Fig. 5A). In the *tt6* mutant line, *C4H* did not show any expression change, but *CHS* showed an ~16-fold increase in expression (Fig. 5B). There was no change in expression levels of the phenylalanine ammonia lyase (*PAL*), 4-coumarate ligase (*4CL*), *CHI*, *CHS*, *DFR*, *F3H*, flavanoid-3′-mono-oxygenase (*F′3H*) leucoanthocyanidin dioxygenase (*LDOX*), and banyuls (*BAN*) genes in transgenic plants (Supplementary Fig. S1 at *JXB* online). In the lignin pathway, *CCR* and *OMT* showed differential expression (Fig. 5A). The decrease in RNA levels of flavonol synthase (*FLS*), *CCR1*, and *OMT1* showed corresponding changes in the metabolite levels in the transgenics. A significant change of 21-fold difference in expression was observed for *CYP98A8*, a cytochrome P450 involved in the phenolamide pathway.

## Discussion

The overall nature of the metabolites and gene expression data in this study provide support that the perturbational effects in the transgenic lines result from *QuoA* expression and are specific in their nature in response to these perturbations. Three transgenic lines selected with low, medium, and high levels of *QuoA* expression exhibited corresponding low, medium, and high levels of *QuoA* enzyme activity, hypocotyl coloration due to anthocyanin accumulation, and stem stiffness due to lignin accumulation, respectively. The overall *QuoA* perturbational effects observed are unlikely to be due to a general stress response. Several stress-related metabolite markers, such as proline, glycine, betaine, and sugar alcohols (Wang *et al.*, 2003), did not show significant changes in QuoA transgenic lines.

Perturbations in the phenylpropanoid network by the expression of bacterial *QuoA* allowed us to gain insights into the relationships between branched pathways. A reduction in the expression of at least 12 genes in the lignin biosynthetic pathway, with the exception of ferulate-5-hydroxylase (*F5H*) (Huang *et al.*, 2009) led to a decrease in lignin content and an increase in various flavonoid metabolites (Hoffmann *et al.*, 2004; Besseau *et al.*, 2007; Li *et al.*, 2008, 2010; Vanholme *et al.*, 2008). The lack of an increase in lignins in the F3H mutant line *tt6* in this study is also consistent with the previous report. In contrast, there is very little known about the reciprocal effects of direct perturbation of the flavonoid pathway on the lignin pathway branch. Simultaneous increases in levels of both anthocyanins and lignins by *QuoA-*mediated perturbation clearly establishes the reciprocal effects of perturbation of flavonoids on the lignin pathway. These relationships between the flavonoid and lignin pathways are likely to differ in various parts of the plant due to their tissue specificity (Grotewold, 2004; Yazaki, 2005) and by the local pools of metabolites being affected by their long-distance transport (Buer *et al*. 2007, 2008). This effect on lignins is also biologically relevant, as shown by the increased stem stiffness of *QuoA* transgenic lines.

One possible explanation of the difference in the perturbational effects between the gain-of-function and loss-of-function approaches here is that intact flavonoid enzyme complexes (Burbulis and Winkel-Shirley, 1999; Saslowsky and Winkel-Shirley, 2001; Conrado *et al.*, 2008) are required for increased channelling of metabolites from flavonoid to lignin pathways. It is quite likely that changes in the levels of anthocyanin intermediates and increases in lignification have resulted from the QuoA gain-of-function approach, where such enzyme complexes would be intact. This raises a possibility whether some of the key enzymes of the lignin pathways are part of the flavonoid metabolic complexes, which can be tested in future.

**Fig. 6.**
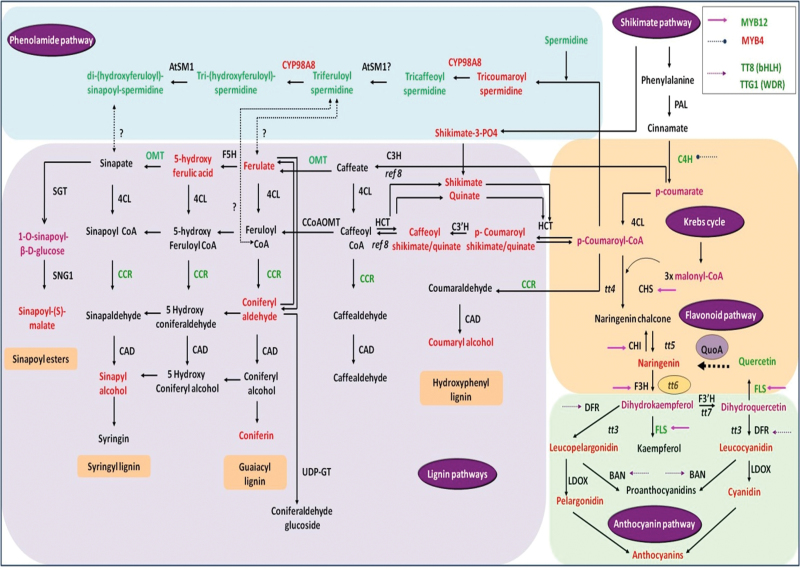
Summary of perturbational effect metabolite and gene expression perturbation resulting from *QuoA* expression. The metabolites that showed overaccumulation and genes that were overexpressed in *QuoA* transgenic plant *Q*10 are indicated in red, while those that were downregulated are shown in green. The coloured arrows indicate the target genes of the regulators that showed differential expression in this study. The key for regulators is shown in the inset box. The point of perturbation is boxed in light orange. Only metabolic pathways belonging to *Arabidopsis* are shown (based on TAIR, http://www.arabidopsis.org/, 10 September 2013; Matsuno *et al.*, 2009; Umezawa, 2009). See Fig. 1 legend for abbreviations. The fold changes are represented as: >2, red; < 2, green; <1.3, purple. All metabolites/genes represented in black fonts did not show changes.

It is intriguing that *QuoA*-mediated perturbation led to upregulation of both lignin and anthocyanin pathways, while the phenylpropanoid pathway above coumaroyl-CoA was unaffected (Fig. 6). Levels of the two members of the upper phenylpropanoid pathway, phenylalanine and cinnamate, were not affected by QuoA perturbation. Thus, this raises the question of which pathway contributes towards simultaneous upregulation of the lignin and anthocyanin pathways? There are no input pathways below the perturbed step in the flavonoid pathway. Hence, the increase in the anthocyanins can only be explained as a result of accumulation of metabolic intermediates from the flavonoid pathway, which then leads to synthesis of anthocyanins. In the case of the lignin pathway, three other input pathways are likely contributors towards its increased levels. These are the upper phenylpropanoid pathway, malonyl-CoA from the Krebs cycle, and the phenolamide pathway (Matsuno *et al.*, 2009; Vogt, 2009; Bassard *et al.*, 2010; Kanehisa *et al.*, 2012). Contributions by the first two pathways cannot explain the increased levels of metabolic intermediates of the lignin pathway, as these were unaffected by the QuoA-induced perturbation. The phenolamide pathway, on the other hand, seems to be a likely candidate for increased levels of metabolites in the lignin pathway. The low, medium, and high expression lines of *QuoA* showed a corresponding effect on the overall reduction of the seven intermediates from the phenolamide pathway. The only exception was tricoumaroyl spermidine, which is associated with the first step in the phenylpropanoid pathway. The F3H mutant *tt6*, which has no increase in lignification, also did not have any perturbational effect on the phenolamide pathway. Lignin upregulation from *QuoA* perturbation, therefore, seems to occur at the expense of phenolamide pathway intermediates. Our study therefore suggests a strong relationship between the lignin and phenolamide pathway and opens up an additional approach to manipulate lignification and stem stiffness characteristics, especially of herbaceous plants.

Our data strongly suggest that the genes and metabolites associated with branch points of pathways have a strong association. Perturbation of metabolite levels was shown here to be associated with changes in the expression levels of genes encoding branch-point enzymes. A complete survey of 17 genes from the four branched pathways showed changes in expression of five genes, all of which were associated with branch points. In each case, the associated products encoded by these genes were also perturbed. The direction of these changes were similar in all cases: while all branch-point genes were downregulated, their corresponding products were upregulated.

Simultaneous analysis of RNA and metabolite levels has uncovered gene–metabolite relationships in several plant models (Holtorf *et al.*, 2002; Stitt *et al.*, 2010). Altered expression levels of cinnamic acid 4-hydroxylase showed the existence of a feedback loop at the entry point into the phenylpropanoid pathway (Blount *et al.*, 2000). In the case of the flavonoid pathway, coumaroyl-CoA has been shown to affect the expression of *PAL*. More recently, flavonoid glycosylation has been shown to be associated with changes in the expression levels of *PAL* (Yin *et al.*, 2012). However, this metabolite-to-gene-expression regulation seems to be a more widespread phenomenon in the overall phenylpropanoid network, as shown here. The biochemical mechanism of control of the gene–metabolite pairs at the branch points is not yet known. Both possibilities remain open: that either there is coordinated control of gene–metabolite pairs via a common mechanism or that there are local regulatory controls at each branch point arising from the common primary perturbational effect. The increase in lignin content with a co-ordinated decrease in the expression of lignin pathway genes also suggests a possible feedback mechanism from lignin or its precursors on gene expression. It will be interesting to investigate the regulatory mechanisms at such branch points and pathways in the metabolic networks.

## Supplementary data

Supplementary data are available at *JXB* online.

Supplementary Fig. S1. Biosynthetic and regulatory genes of phenylpropanoid pathway, whose expressions did not change due to QuoA perturbation.

Supplementary Table S1. Bacterial strains, plasmids, and plant types used in this investigation and their features.

Supplementary Table S2. Reverse transcription PCR and quantitative real-time PCR primers.

Supplementary Table S3. Lysine intensity levels in the *Arabidopsis* lines used in this study.

Supplementary Table S4. Intensity levels of lysine and phenolics in the *Arabidopsis* lines used in this study.

Supplementary Table S5. Metabolites and their fragment ions that were used to quantify level of metabolites through the multiple reaction monitoring approach.

Supplementary Table S6. Standard metabolites used in the study and their MS/MS peaks.

Supplementary Data

## Abbreviations:

ESI: electrospray ionization
GUS: β-glucuronidase
HPLC: high-performance liquid chromatography
MRM: multiple reaction monitoring
MS: mass spectrometry
WT: wild type
