# Supplementary material for: A bacterial quercetin oxidoreductase QuoA-mediated perturbation in the phenylpropanoid metabolic network increases lignification with a concomitant decrease in phenolamides in Arabidopsis
Source: J Exp Bot. 2013 Oct 1;64(16):5183–94. doi: 10.1093/jxb/ert310 (PMC3830493; doi:10.1093/jxb/ert310)
Supplement: Supplementary Data [file supp_64_16_5183__index.html]

A bacterial quercetin oxidoreductase QuoA-mediated perturbation in the phenylpropanoid metabolic network increases lignification with a concomitant decrease in phenolamides in Arabidopsis — A bacterial quercetin oxidoreductase QuoA-mediated perturbation in the phenylpropanoid metabolic network increases lignification with a concomitant decrease in phenolamides in Arabidopsis — Supplementary Data 

# A bacterial quercetin oxidoreductase QuoA-mediated perturbation in the phenylpropanoid metabolic network increases lignification with a concomitant decrease in phenolamides in *Arabidopsis*

## Supplementary Data

Data files

**Files in this Data Supplement:**

- Supplementary Data - Supplementary Data
