# Supplementary material for: A bacterial quercetin oxidoreductase QuoA-mediated perturbation in the phenylpropanoid metabolic network increases lignification with a concomitant decrease in phenolamides in Arabidopsis
Source: J Exp Bot. 2013 Oct 1;64(16):5183–94. doi: 10.1093/jxb/ert310 (PMC3830493; doi:10.1093/jxb/ert310)
Supplement: Supplementary Data [file supp_ert310_jexbot103424_file001.pdf]

A bacterial quercetin oxidoreductase QuoA-mediated perturbation in phenylpropanoid metabolic network increases lignification with concomitant decrease in phenolamides in *Arabidopsis*

Sheela Reuben, Amit Rai, Bhinu Pillai, Amrith Rodrigues, and Sanjay Swarup

Supplementary Data

Supplementary Data

A. B.

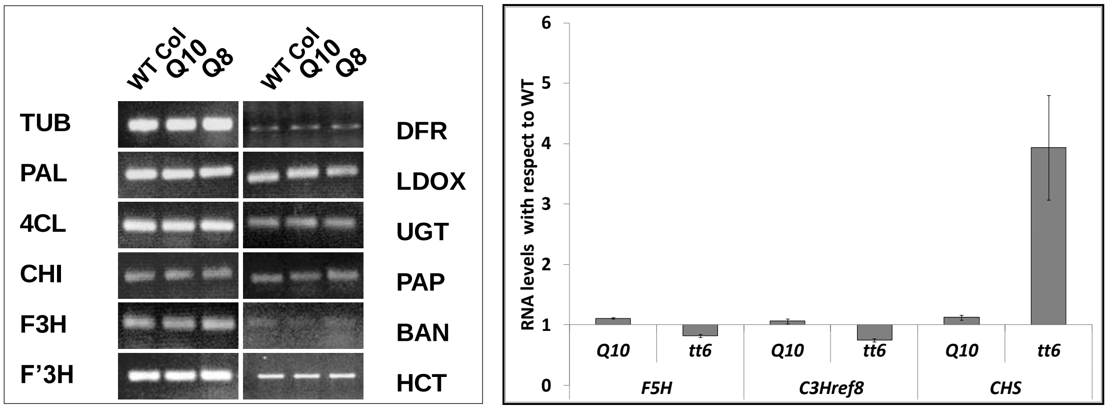

Supplementary Figure 1: Biosynthetic and regulatory genes of phenylpropanoid pathway, whose expressions did not change due to QuoA perturbation. *TUB*: β-Tubulin, PAL: Phenylalanine ammonia lyase, *4CL*: 4-coumaroyl-coenzyme A ligase, *CHI*: Chalcone-flavanone isomerase, *F3H*: Flavanone 3-hydroxylase, *F3'H*: Flavonoid 3'monooxygenase, *C4H*: Cinnamate 4-hydroxylase, *DFR*: Dihydroflavonol 4-reductase, LDOX: Leucoanthocyanidin dioxygenase, *UGT*: UDP glucose transferase, PAP: Production of anthocyanin pigment; *BAN*: Banyuls *HCT*: Hydroxycinnamoyl-CoA shikimate/quinate hydroxycinnamoyl transferase, *F5H*: Ferulate 5-hydroxylase, *C3H*(ref8): Coumarate-3-hydroxylase (*ref8*). *CHS*: Chalcone synthase shows 3-fold change in *tt6* but no change in transgenic Q10.

Supplementary Table 1: Bacterial strains, plasmids and plant types used in this investigation and their features.

Strain or plasmids	Genotype or phenotype[a]	Source or reference
Strains		
P. putida		
PML2	Spontaneous Cm^r mutant of a plant growth promoting rhizobacterial ATCC39270 strain. Utilizes quercetin, naringenin, daidzein, apigenin, hespertin and naringin	(Pillai & Swarup, 2002)
Quo^- mutant1	mini-Tn5*gfp* mutants of PML2, quercetin negative, naringenin negative, Cm^r, Gm^r, Km^r. *Php* is now renamed as *Quo* (quercetin oxidation). Same as Php^- mutant1.	(Pillai & Swarup, 2002)
PML2pGB*QuoA*	Quo- mutant Cm^r, Gm^r, Km^r, Tc^r,contains pGB*QuoA*.	This study
PML1	Spontaneous Cm^r, Rf^r, mutant of a plant growth promoting rhizobacterial ATCC39169 strain. Unable to utilize any of the phenylpropanoid compounds	(Pillai & Swarup, 2002)
PML1pGB*QuoA*	PML1 strain Cm^r, Rf^r, Tc^r, contains pGB*QuoA*. Utilizes quercetin	This study
E. coli		
SM10 (λpir)	*pro thi-1 thr-1 leuB6 supE44 tonA21 hsdR⁻M⁺ lacY1 recA* RP4-2-Tc:Mu-Km, λpir), a derivative of *E. coli* C600, conjugative donor of mini-Tn*5*	(Pillai & Swarup, 2002)
DH5α	*supE44*Δ (*lacU169*) (ϕ80lacZΔM15) *hsdR17 recA1 endA1 gyrA96 thi-1 relA1* F^-	Gibco-BRL
A. tumefaciens		
AGL1	Derived from AGL0, *recA*::*bla*, C58, Hypervir, Onc^-, pTiBo542(delta)T, Mop^+, Cb^r, Km^r, Rf^r	Lazo *et al.*, 1991
Plasmids		
pAG408	Carries mini-Tn5*gfp*, Km^r, Gm^r, Ap^r, *tnp*, RP4 *oriT*, R6K *oriV*	Suarez *et al.*, 1997
pGEM®-3Zf(+)	Cloning vector, f1 ori for + strand	Promega Corp
pDrive	TA cloning vector, Ap^r	Qiagen
pRTL2GUS	Transient plant expression vector, containing GUS reading frame with in *Nco*I and *BamH*I sites, duplicated CaMV35S promoters, Tobacco Etch Virus (TEV) enhancer, polyA tail, CaMV terminator	Carrington *et al.*, 1991
pRTL2QuoA	PRTL2 carrying 2.2-kb QuoA fragment in place of GUS	This study
pGB1	pUCP18 containing 1.8-kb fragment from pWTT2081 carrying Tc^r	Bloemberg *et al.*, 1997
pGB*QuoA*	pGB1 carrying *QuoA*, in-frame ORF	This study
pBIN20	Plant transformation binary vector, Km^r, 35S promoter, also containing Km^r between LB and RB	Hennegan and Danna, 1998
pBin*QuoA*	pBIN20 carrying *Hind*III-*Hind*III fragment containing *QuoA* expression cassette, dual CaMV-35S promoters, polyA, CaMV-terminator	This study
Plant ecotypes		
tt6/ler	F3H *Arabidopsis* mutant (Germplasm Name-CS87)	ABRC
wt/ler	Wild type *Arabidopsis* landsberg ecotype (Germplasm Name-CS20)	ABRC
wt-QuoA/col	Transgenic *Arabidopsis* plants expressing microbial *QuoA* gene	This study
wt/col	Wild type *Arabidopsis* Columbia ecotype (Germplasm Name-CS60000)	ABRC

ABRC: Arabidopsis Biological Resource Center, USA. Ap, ampicillin; Cb, carbenicillin; Cm, chloramphenicol; Rf, rifampicin, Gm, gentamycin; Km, kanamycin; Tc, tetracycline; r(superscript), resistance; Mop, mannopine utilization; pTi, "overdrive" sequence; Onc, oncogenicity.

Supplementary Table 2: Reverse transcriptase polymerase chain reaction and quantitative real time PCR primers.

**Reverse Transcriptase Polymerase Chain Reaction Primers**		
**Biosynthetic Gene Primers**		
TUB-P1	5'ATCCGTGAAGAGTACCCAGAT3'	β tubulin
TUB-P2	5'TCACCTTCTTCATCCGCAGTT3'	β tubulin
PAL1FP	GGAGGAGTGGACGCTATGTTATG	Phenylammonia lyase 1 (AT2G37040)
PAL1RP	GGAGTGTGGCAATGTGTGGC	Phenylammonia lyase 1 (AT2G37040)
4CL1FP	CGTCGTCATGCTCCTCCTCC	4 coumarate ligase (AT1G51680)
4CL1RP	CGCTCGTGACTAGTCCCTTGTGA	4 coumarate ligase (AT1G51680)
C4HFP	TGTTCACTGTTTACGGCGAGC	Cinnamic acid 4-hydroxylase(AT2G30490)
C4HRP	CGGTCCAAGAACTGTGTCGAG	Cinnamic acid 4-hydroxylase(AT2G30490)
CHSFP	5'GCAGGCATCTTGGCTATTG3'	Chalcone synthase (AT5g13930)
CHSRP	5'AGTCTGGAAGGATGGTCTGAG3'	Chalcone synthase (AT5g13930)
CHIFP	CACCGTCATTGGAGTATACCTAGAG	Chalcone isomerase (AT3G55120)
CHIRP	TCATTCTCAGTTCTCTTTGGCTAGT	Chalcone isomerase (AT3G55120)
DFRFP	5'GATCCTGAGAACGAAGTGA3'	Dihydroflavonol 4-reductase (AT5g42800)
DFRRP	5'CATTTCCTCGAGACTATACTT3'	Dihydroflavonol 4-reductase (AT5g42800)
F3Htt6FP	GTGACGGAGGAGTATAGTGAGAGG	Flavanone 3-hydroxylase (AT3G51240)
F3Htt6RP	CCATCTTTCTCTTATACATCTCGG	Flavanone 3-hydroxylase (AT3G51240)
F3'Htt7FP	TGGTGACGGAAATGATGGCTC	Flavonoid 3'-hydroxylase (AT5G07990)
F3'Htt7RP	GATGTGTGGTAACGAGAGTGGTGT	Flavonoid 3'-hydroxylase (AT5G07990)
FLSFP	CAGGGAGGTGAATGAAGAGTATGC	Flavonol synthase (AT5G08640)
FLSRP	GCTTGCGGTAACTGTAATCCTTG	Flavonol synthase (AT5G08640)
LDOXFP	GGTCCTCAAGTTCCCACAATCG	Leucoanthocyanidin dioxygenase (AT4G22880)
LDOXRP	GTGACCCATTTGCCCTCGTAG	Leucoanthocyanidin dioxygenase (AT4G22880)
BANFP	GACTCTTACACACACCGGATCG	BAN (AT1G61720)
BANRP	CGGAGGATCGGAGAGGAGAG	BAN (AT1G61720)
UDPgluFP	GCAGAACCAACTTAACCATCCTC	UDP glucose transferase (AT3G21560)
UDPgluRP	GAGTGGTCCTAGCGGTCTGATAAC	UDP glucose transferase (AT3G21560)
C3HFP	GCACCGGAACAGATCGACG	Coumarate -3-hydroxylase (AT2G40890)
C3HRP	CCTAGCTTCAGACCGTTGGATACT	Coumarate -3-hydroxylase (AT2G40890)
HCTFP	5'CCACCGAGACACCAATCA3'	Shikimate/quinate O-hydroxycinnamoyltransferase (AT5g48930)
HCTRP	5'AAGTAACCAGGAGGCAGCT3'	Shikimate/quinate O-hydroxycinnamoyltransferase (AT5g48930)
**Regulatory Gene Primers**		
PAP1FP	GGCACCAAGTTCCTGTAAGAGC	PAP1 (AT1G56650)
PAP1RP	GGCATGGAGGATTAACGTCAAC	PAP1 (AT1G56650)
AtMYB4FP	GGGAACAACTCAACTACTGCAACAC	Atmyb4 (AT5G26660)
AtMYB4RP	CTTAGCACCAAAAAAATTCACTCGAG	Atmyb4 (AT5G26660)
MYB12FP	5'ACGTCTGCACCACCGGAG3'	MYB12 (AT2g47460)
MYB12RP	CCAAACACAATCCCAATCG	MYB12 (AT2g47460)
tt8FP	CTGAACACTCCACCTACGAAG	tt8 (AT4G09820)
tt8RP	CTTGCTGATGCTCTTTGCC	tt8 (AT4G09820)
ttgFP	TGGCTACGATTTTGATGGATTC	ttg (AT5G24520)

ttgRP	CAGATAGATACAGAGTCATTGCGG	ttg (AT5G24520)
**Reverse transcriptase Quantitative Real Time Polymerase Chain Reaction Primers**		
**Biosynthetic Gene Primers**		
Tub FPRealT	CCGGAGCTGACACAGCAAAT	β tubulin -TUB1(AT1G75780)
Tub RPRealT	GTCCGTGGCGTGGATCA	β tubulin-TUB1(AT1G75780)
PALRealTFP	CATTGTATAGATTCGTGAGGGAAGAG	Phenylammonia lyase 1 (AT2G37040)
PALRealTRP	TCCAGGCGACGTCACTTTCT	Phenylammonia lyase 1 (AT2G37040)
C4HFPReal	TCCTCGTGCCTCACATGAAC	Cinnamic acid 4-hydroxylase(AT2G30490)
C4HRPReal	TGCTTTCTGCTGGGATATCGT	Cinnamic acid 4-hydroxylase(AT2G30490)
CHS FPRealT	CTCATGTCGTCTTCTGCACTACCT	Chalcone synthase (AT5g13930)
CHS RPRealT	GAAGCTTGGTGAGCTGGTAGTCA	Chalcone synthase (AT5g13930)
DFR FPRealT	AACATTGGTGGTCGGTCCAT	Dihydroflavonol 4-reductase (AT5g42800)
DFR RPRealT	GATAGGAGAGAGCGCGGTGAT	Dihydroflavonol 4-reductase (AT5g42800)
F3HRealTFP	GGAGCGTTTGTCGTCAATCTC	Flavanone 3-hydroxylase (AT3G51240)
F3HRealTRP	CAGCATTCTTGAACCTCCCATT	Flavanone 3-hydroxylase (AT3G51240)
F3'HRealTFP	CGTGGTCGCCGCTTCTAA	Flavonoid 3'-hydroxylase (AT5G07990)
F3'HRealTRP	CGAAATTGGCGTCGTGTATTT	Flavonoid 3'-hydroxylase (AT5G07990)
FLSRealTFP	TCACAACATTCCGAGGTCCAA	Flavonol synthase (AT5G08640)
FLSRealTRP	CTTCGTCGGGATCGCTTAGA	Flavonol synthase (AT5G08640)
C3Href8FP1Real	CCGCAACGGTCAGGATCT	Coumarate -3-hydroxylase (AT2G40890)
C3Href8RP1Real	GCGTGCAAACTTTTCTCACCTT	Coumarate -3-hydroxylase (AT2G40890)
C3HFP1Real	CGCGGGTGCTGACACAA	At1g74540 putative C3H now annotated as Cyt p450
C3HRP1Real	CACGGTCGGGCATTTGAT	At1g74540 putative C3H now annotated as Cyt p450
CCR1FPReal	TGCAAAAACACCAAGAATTGGT	Cinnamoyl-CoA reductase (AT1G15950)
CCR1RPReal	TCTCCCACGCCGCTTGT	Cinnamoyl-CoA reductase (AT1G15950)
F5HFPReal	GAGTGGGCCTTAACGGAGTTATT	Ferulate 5 hydroxylase (AT4G36220)
F5HRPReal	TCGGCGAGTTCTTGTTGGA	Ferulate 5 hydroxylase (AT4G36220)
ATOMTFPReal	CTTAACCTGCTCCAACCGTAAACT	Flavonol 3'-O-methyltransferase (AT5G54160)
ATOMTRPReal	CCGGACCAAGCCCGTAA	Flavonol 3'-O-methyltransferase (AT5G54160)
**Regulatory Gene Primers**		
MYB12 FPRealT	AAAAACTCGTAAAACGAAGAAAACG	MYB12 (AT2g47460)
MYB12 RPRealT	TCTTTATCAGCCCCAGCTACATC	MYB12 (AT2g47460)
tt8 FPRealT	CCAACAGCTCAGGGAGCTTT	tt8 (AT4G09820)
tt8 RPRealT	GCGGTGCATGCTCTTGCT	tt8 (AT4G09820)
AtMYB4RealTFP	TCGCAACAAGGTCCATGGT	Atmyb4 (AT5G26660)
AtMYB4RealTRP	AAAGCAAAACGCAGAGTTTTGAT	Atmyb4 (AT5G26660)
ttgRealTFP	CAACAGCAAAACGAGCGAGTT	ttg1 (AT5G24520)
ttgRealTRP	GGCTCTACATCGTTCCAATCG	ttg1 (AT5G24520)

FP: Forward Primer; RP: Reverse Primer

Supplementary Table 3: Lysine intensity levels in the *Arabidopsis* lines used in this study.

Arabidopsis Lines	Average and SD of raw intensity value for lysine (CPS)	% change w.r.t Ler	p-value of pair-wise means comparison with Ler	Coefficient of variation (%)
Ler	4457891±5094.89	0	0.039	0.133
tt6	4476880±4424.55	0.42	0.01	
Col-0	4431881±3575.11	-0.58	0.043	
Q10	4429890±4238.77	-0.63	0.029	
Q2	4431098±3836.11	-0.60	0.037	
Q11	4427998±4869.44	-0.67	0.019	

SD denotes standard deviation

Supplementary Table 4: Intensity levels of lysine and phenolics in the *Arabidopsis* lines used in this study.

	**Average raw intensity values (CPS)**							
	**Col-0**	**Q2**	**Q10**	**Q11**	**Ler**	**tt6**	**SD**	**Coefficient of variation (%)**
lysine (control)	*4431881*	*4431098*	*4429890*	*4427998*	*4457891*	*4476880*	*18397.86*	*0.41*
cyanidin	161140	343833	841166	407450	153660	138475	245985.07	72.15
pelargonidin	84166	411725	1104916	149150	242875	300875	339693.95	88.86
quercetin	237875	158400	134916	187366.66	165375	204575	33438.17	18.43
leucopelargonidin	248100	545150	4020966	129600	185700	4300	1425104.15	166.55
cyanidin 3-monoglucoside	359250	521740	1307100	785133.34	437000	322925	341256.20	54.85
naringenin	457450	6086325	9001150	930966.66	312283	281033	3434066.38	120.71
ferulate	541700	955916	2104133	684733.33	259040	284150	627650.40	77.97
coumaryl shikimate	511975	8127760	9896150	172983.33	456650	441450	4095068.48	125.31
sinapyl alcohal	583980	1262840	3063766.67	815983.33	525883	299466	930942.02	85.25
sinapoyl-s-malate	805760	2415550	4096600	316850	757266	563240	1347294.58	90.27
coumaryl alcohal	1231120	2849250	7046716	1720200	1440883	724850	2132510.44	85.23
tricoumaroyl spermidine	1201280	2108666	729500	692383.35	1418216	621600	525405.23	46.55
tricaffeoyl spermidine	1941340	970800	107400	562066.7	1461716	1150460	592319.62	57.38

SD denotes standard deviation

Supplementary Table 5: Metabolites and their daughter ions that were used to quantify level of metabolites through multiple reaction monitoring approach.

**Metabolite**	**M/Z**	**Daughter ions**
3-dehydroshikimate	173.14	61.1, 113.1, 75.1
3,7-di-O-methylquercetin	331.3	137, 165, 229, 201
4-coumaric acid	165.047	119, 65, 77, 91
4-coumaroylquinate	339.3	95, 77, 129, 87.1
5-enolypyruvil shikimate 3 phosphate	325.18	71, 87, 61.1
caffeate	181.0423	117.03, 135.04, 145.02
caffeoyl shikimate	337.29	163.03, 95.1, 117.03, 89.03
chorismic acid	225.17	70, 86
coffeoyl CoA	930.68	163.03, 145.02
coffeoyl quinate	355.3	89.03, 95, 117.03, 163.03
coniferyl alcohol	181.078	99, 140
coumarine	147.03	65, 91, 60
coumarinic acid	327.2421	77, 103, 165.02,
coumaryl skhimiate	151.17	91, 77, 61.1, 95.1
cyanidin	288.05	121.02, 109.02, 157.06
delphinidin	302.23	137, 165, 153
dihydroxyferuloyl sinocoyl spermidine	736.79	146.2, 147.04, 193, 175.044
ferulatic acid	195.06	89, 177, 117
N3 tricaffeoyl spermidine	632.27	146.2, 163.04, 89.03, 135.0441
N3 tricoumaroyl spermidine	584.36	146.2, 103, 112.1,
naringenin	273.07	91.054, 123.044, 153.018, 119.05
4-courmaryl CoA	914.67	103, 165.02, 221.18
quinnic acid	193.06	87.1, 95, 129
shikimate	175.06	75.1, 101.1, 61.1, 95.1
shikimate 3 phosphate	255.13	95.1, 113.1, 101.1
sinapic acid	225.07	119.05, 147.048, 91.05, 175.044
sinapoyl-s-malate	341.25	175.044, 207.07, 147.048, 119.05
spermidine	146.2	72.1, 84.1, 58.1, 112.1
transcinnamic acid	149.05	105, 59.4, 79

triferuloyl spermidine	674.76	146.2, 177, 89, 117
trihydroxyferuloyl spermidine	722.76	146.2, 193

Supplementary Table 6: Standard metabolites used in the study and their MS/MS peaks.

**Metabolite**	**Molecular ion**	**MS/MS peaks**
Cyanidin chloride	323.7	72.8, 90.9, 108.8, 114, 266.9
Ferulic acid	195.07	98.7, 99.1, 194.8, 117.2
Caffeic acid	181.1	144.9, 103.8, 62.8, 99,63
Naringenin	273.07	153.0, 118.9, 90.7
Quercetin	303.04	131, 158.8, 161, 169.2, 172.9, 283.8
Spermidine	146.23	55.1, 59.96, 72.1, 75.2, 84.1, 112.1, 129.1
